# Effect of Compound Danshen Injection Combined with Magnesium Sulfate on Oxidative Stress, TNF-*α*, NO, and Therapeutic Efficacy in Severe Preeclampsia

**DOI:** 10.1155/2022/9789066

**Published:** 2022-07-18

**Authors:** Yanling Zhou, Juan Wang, Lei Wang, Jing Tang, Chengwei Zhang

**Affiliations:** ^1^Department of Obstetrics, Yantaishan Hospital, Yantai 264000, China; ^2^Department of Clinical Laboratory, Zhangqiu District People's Hospital, Jinan 250200, China; ^3^ICU, The Affiliated Qingdao Central Hospital of Qingdao University, The Second Affiliated Hospital of Medical College of Qingdao University, Qingdao 266042, China; ^4^Department of Gynaecology, Zhangqiu District Maternity and Child Care Hospital, Jinan 250200, China; ^5^Medical Laboratory and Diagnostic Center, Jinan Central Hospital, Jinan 250013, China

## Abstract

**Aims:**

This study is designed to explore the effect of compound Danshen injection combined with magnesium sulfate on TNF-*α*, NO, oxidative stress, and therapeutic efficacy in severe preeclampsia (S-PE).

**Methods:**

Sixty S-PE patients were placed into the control group and the therapy group, randomly. The control group was under the treatment of magnesium sulfate, and the therapy group was under the treatment of compound Danshen injection with magnesium sulfate. After treatment, the therapeutic efficacy of the two groups was comparatively analyzed.

**Results:**

7 days after treatment, DBP, SBP, and 24 h urinary protein were sharply lower than those before treatment. The 24 h urinary protein was notably lower in the therapy group. After treatment, the expression level of TNF-*α* in both groups was notably higher than before treatment, while NO level was higher than that before treatment. Furthermore, D-D level in two groups was dramatically decreased compared to that before treatment. Moreover, Fib, PT, and APTT in two groups showed statistically significant differences after 7 days. The contents of ALT, AST, BUN, and Scr in therapy group were notably lower than those in control group.

**Conclusion:**

Our results indicated that compound Danshen injection could improve renal function, blood hypercoagulability, and oxidative stress level and had a better therapeutic effect on S-PE.

## 1. Introduction

Preeclampsia (PE) is a unique disease of women during pregnancy. The main clinical manifestations are hypertension, edema, proteinuria, and other symptoms after 20 weeks of pregnancy [1–4], causing systemic multi-system dysfunction and even irreversible damage in pregnant women [5–7] and seriously threatening maternal and infant health. The incidence of PE is about 5% [8, 9]. Therefore, timely and effective treatment is of significant meaning to the prognosis of PE patients. At present, the pathogenesis of PE is not completely clear [10], among which the theory of placental shallow implantation and the theory of vascular endothelial injury are the main theories. As the core factor of inflammatory response, excessive TNF-*α* can directly damage vascular endothelial cells and cause placental shallow implantation, placental ischemia, and hypoxia [11–14]. NO acts as an endogenous vasodilator released by endothelial cells. When its synthesis, release, and activity are inhibited and destroyed, it will further lead to abnormal vasoconstriction and dysfunction, which will worsen local or systemic ischemia and hypoxia and worsen the condition of patients with severe PE (S-PE) [15]. Compound Danshen injection has the effects of promoting blood circulation, preventing platelet aggregation, removing free radicals, protecting vascular endothelial cells, and improving cell resistance to hypoxia [16, 17]. Studies [18, 19] have shown that compound Danshen injection has certain therapeutic efficacy in treating S-PE. However, it has not been reported whether compound Danshen injection can affect the changes of clinical parameters such as blood pressure by reducing TNF-*α* and increasing NO level.

Magnesium sulfate is the drug of choice for relieving spasm of S-PE, which can relax skeletal muscle and effectively control the patient's central nervous system. Although the therapeutic effect of magnesium sulfate has been recognized by clinical trials, excessive blood concentration of the drug may cause dizziness, nausea and vomiting, abdominal pain and diarrhea, and other suspected mild poisoning [20, 21], which threatens the safety of patients to a large extent. Therefore, magnesium sulfate often needs to be used in conjunction with other drugs to reduce the degree of adverse reactions and improve the efficacy. In this study, compound Danshen injection was used to treat S-PE patients, in order to observe the clinical efficacy and the effect on TNF-*α* and NO.

## 2. Materials and Methods

### 2.1. Research Objects

60 patients with S-PE who underwent regular prenatal examination and were hospitalized in the department of obstetrics of our hospital were selected. Patients were placed into therapy group and control group. 30 patients in control group were treated with magnesium sulfate. 30 patients in therapy group were under the treatment of compound Danshen injection on the basis of control group. All pregnant women signed the informed consent form.

### 2.2. Diagnostic Criteria for S-PE

- Systolic blood pressure (SBP) ≥160 mmHg and/or diastolic blood pressure (DBP) ≥110 mmHg
- Proteinuria (++) and above
- Serum creatinine (Scr) ≥ 1.2 mg/dL (1 mg/dL = 88.402 *μ*mol/L)
- Platelet (Plt) < 100×10^9^/L

S-PE can be diagnosed with any of these laboratory results.

### 2.3. Inclusion and Exclusion Criteria

Inclusion criteria were as follows:

- Met the diagnostic criteria of moderate to S-PE in Obstetrics and Gynecology
- No previous history of PE
- The gestational age of onset <34 weeks, and the gestational age of delivery was 28–33 weeks
- No other treatment was received before admission
- Normal basic coagulation function before pregnancy
- Full participation in treatment and related examinations and complete clinical data
- Signed informed consent and approved by the medical ethics committee of our hospital

Exclusion criteria were as follows:

- Complicated with severe cardiac, hepatic, and renal insufficiency
- With diabetes, hypertension, and other chronic diseases [22]
- With malignant neoplastic diseases
- Related treatment contraindications
- Communication disorders
- Poor compliance

### 2.4. The Treatment

Patients in both groups were on bed rest, their fetal heart rates were closely monitored, and they were given oxygen inhalation. Routine examinations such as electrocardiogram, metabolic indicators, renal function indicators, coagulation indicators, and electrolytes were performed.. The control group was under the conventional treatment and magnesium sulfate. 5 g magnesium sulfate was dissolved in 20 mL 5% glucose solution and dropped within 30 minutes. Then, 15 g magnesium sulfate was dissolved in 500 mL 5% glucose solution and dropped for 2 hours. The total amount of magnesium sulfate used for 24 hours should not exceed 30 g. One course of treatment is one week.

The therapy group was treated with compound Danshen injection in addition to the basic treatment of the control group. Compound Danshen injection (16 ml) was injected into 250 ml 5% glucose solution, once a day.

### 2.5. Evaluation Criteria for Clinical Effect

1. Cured: the patient's clinical symptoms disappear, blood pressure <140/90 mmHg, and no symptoms of proteinuria or edema
2. Effective: the patient's clinical symptoms improved and blood pressure was 140/90 mmHg to 150/100 mmHg, with slight proteinuria and slight edema
3. Ineffective: the patient's symptoms did not improve, and the condition was gradually transformed from mild PE to S-PE

Total effective rate = ((cured + effective)/total number of cases) × 100%.

### 2.6. Detection of TNF-*α*

Enzyme-linked immunosorbent assay (ELISA) was used for TNF-*α* detection. The experiment was carried out in the laboratory of our hospital, and all operation procedures were strictly carried out according to the product instructions.

### 2.7. Detection of NO

The nitric acid reductase method was used to detect NO in serum. This experiment was completed in the laboratory of our hospital, and each experiment was conducted in strict accordance with the instructions.

### 2.8. Detection of Endothelial Protein C Receptor (EPCR) and Thrombomodulin (TM)

The levels of EPCR and TM were examined by ELISA before and after treatment.

### 2.9. Detection of Lipid Peroxide (LPO)

The catalase (CAT) level was determined by ammonium molybdate colorimetry, and the LPO level was determined by thiobarbituric acid colorimetry before and after treatment.

### 2.10. Coagulation Function

Before and after 7 days of treatment, 1.5 ml of fasting venous blood was taken from both groups and added with sodium citrate for anticoagulation. The serum was extracted by centrifugation at 3000 r/min at 4°C for 10 min. The levels of fibrinogen (Fib), prothrombin time (PT), D-dimer (D-D), and activated partial thrombin time (APTT) were determined by coagulation analyzer.

### 2.11. Liver and Kidney Function

The contents of liver and kidney function indexes in peripheral blood, including blood urea nitrogen (BUN), aspartate aminotransferase (AST), blood creatinine (Scr), and alanine aminotransferase (ALT), were detected by automatic blood biochemical analyzer.

### 2.12. Statistical Analysis

The measurement data were presented as $\left(\bar{x}+s\right)$ and analyzed by *t*-test. The numeration data were presented as N (%), and analyzed by *χ*^2^ test. SPSS 22.0 software was used for data analysis. The *t*-test was applied to data analysis between groups. When *p* < 0.05, the difference was considered statistically significant.

## 3. Results

### 3.1. Comparison of Clinical Parameters between the Two Groups before Treatment

The age of patients in the therapy group was 24–38 years old, the average age was 28.6 ± 4.9 years old, the gestational age was 28–33 weeks, and the average gestational age was 29.90 ± 2.40 weeks. There were 22 cases of cesarean section and 8 cases of vaginal delivery. Patients in the control group were 25–39 years old, with an average age of 29.1 ± 4.8 years old. The gestational age of pregnant women was 28–33 weeks, with an average gestational age of 29.70 ± 2.80 weeks. There were 23 cases of cesarean section and 7 cases of vaginal delivery. Before treatment, there was no obvious difference in parameters between the two groups (*p* > 0.05).

### 3.2. Comparison of TNF-*α* and NO between the Two Groups

**Figure 1:**
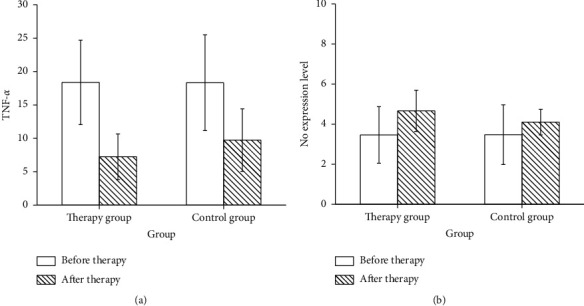
Comparison of TNF-*α* and NO levels between two groups. (a) Comparison of TNF-*α* before and after treatment. (b) Comparison of NO before and after treatment.

There was no significant difference in TNF-*α* level between the two groups before treatment. TNF-*α* level in two groups was evidently reduced after treatment, and the decrease was more obvious in therapy group. The level of TNF-*α* in the two groups was notably different after 7 days (*p* < 0.05, Figure 1(a)).

There was no obvious difference in NO level between the two groups prior treatment. The level of NO in the control group increased sharply after treatment. Also, the level of NO in the therapy group increased more obviously after treatment than before treatment. The difference of NO level between the two groups after treatment was statistically significant (*p* < 0.05, Figure 1(b)).

### 3.3. Comparison of EPCR and TM Levels between the Two Groups

There was no obvious difference in EPCR and TM levels between the two groups before treatment (*p* > 0.05). After treatment, the levels of EPCR and TM in two groups were lower than before treatment (*p* < 0.05). The levels of EPCR and TM in the thearpy group were lower than those in the control group (*p* < 0.05, Table 1).

### 3.4. Comparison of CAT and LPO Levels between the Two Groups

Before treatment, there was no statistical significance in CAT and LPO levels between the two groups (*p* > 0.05). After one week of treatment, CAT level in both groups was higher (*p* < 0.05), LPO level was lower than that before treatment, and the range of change in the therapy group was greater than that in the control group (*p* < 0.05, Table 2).

### 3.5. Comparison of Coagulation Function Indexes before and after Treatment

Before treatment, there were no evident differences in serum coagulation indexes Fib, D-D, PT, and APTT between two groups (*p* > 0.05). The levels of Fib and D-D in two groups after treatment were lower, while PT and APTT levels were higher than those before treatment (*p* < 0.05). The levels of Fib and D-D in the therapy group were lower, while the levels of PT and APTT were higher than those in the control group (*p* < 0.05, Table 3).

### 3.6. Comparison of Liver and Kidney Function Indexes

Before receiving treatment, there was no difference in the levels of ALT, AST, BUN, and Scr between two groups (*p* > 0.05). After 7 days of treatment, the levels of ALT, AST, BUN, and Scr in two groups were lower than those before treatment (*p* < 0.05). The levels of ALT, AST, BUN, and Scr in the therapy group were lower than those in the control group (*p* < 0.05, Table 4).

### 3.7. Comparison of Clinical Effective Rate between the Two Groups

**Figure 2:**
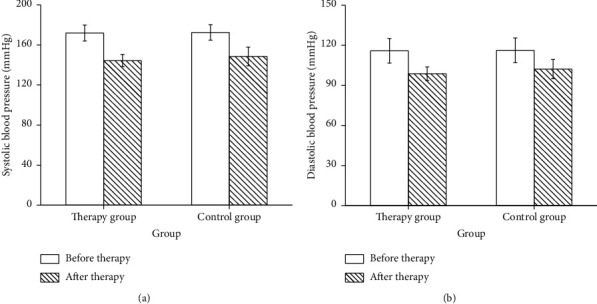
Comparison of blood pressure levels between two groups before and after treatment. (a) SBP. (b) DBP.

**Figure 3:**
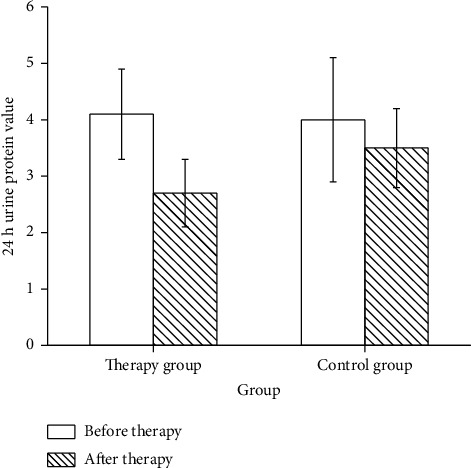
Comparison of 24 h urine protein between two groups before and after treatment.

**Figure 4:**
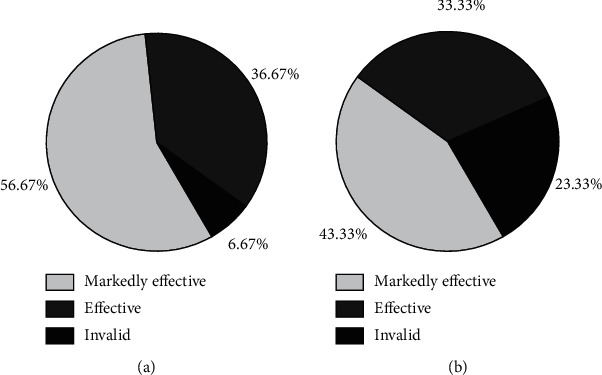
Comparison of clinical effective rate between the two groups. (a) Effective rate of therapy group. (b) Effective rate of control group.

Before treatment, there were no differences in SBP, DBP, and 24 h urine protein between two groups (*p* > 0.05). Compared with before treatment, blood pressure levels in the two groups declined after treatment, and the decrease was more obvious in the treatment group. After treatment, there were notable differences in blood pressure levels between the two groups (*p* < 0.05, Figure 2). After 7 days of treatment, the 24 h urinary protein in the therapy group was dramatically lower than that in the control group (*p* < 0.05, Figure 3). The total effective rate in the therapy group was 93.0%, and the total effective rate in the control group was 76.7%. The overall effective rate of the therapy group was maintained at a high level (*p* < 0.05, Figure 4).

## 4. Discussion

S-PE is mainly manifested by continuous elevated blood pressure and renal impairment and may also be accompanied by persistent headache, visual impairment, epigastric pain,, and other clinical symptoms, which bring discomfort to pregnant women and directly threaten the safety of the lives of mothers and infants [7, 23]. Conventional treatments such as hypotension, spasmolysis, and sedation have been proved to be effective for mild PE, but the outcome optimization effect of the above methods on S-PE is limited. Compound Danshen injection has been used for the treatment of acute myocardial infarction and angina pectoris, and its effective component tanshinone can protect the myocardium, remove oxygen free radicals, and improve blood flow. Pregnant women with S-PE have some pathological manifestations similar to myocardial infarction, such as systemic arteriolar spasm, placental atherosclerosis, and decreased blood perfusion. Therefore, compound Danshen injection was added into the treatment plan of pregnant women with S-PE as an auxiliary drug.

The study showed that TNF-*α* in both groups was decreased after treatment, and the decrease was more obvious in the therapy group. The lower the TNF-*α* level, the higher the probability of S-PE disease improvement, suggesting that compound Danshen injection combined may play a certain effect on PE patients by reducing the TNF-*α* level. The level of NO in both groups increased after treatment, and the increase was especially significant in the therapy group. The higher the NO level is, the stronger the S-PE could be controlled, suggesting that magnesium sulfate combined with compound Danshen injection may play a therapeutic role in S-PE by increasing the level of NO. Therefore, it can be inferred that compound Danshen injection is related to reducing TNF-*α* and increasing NO level.

The blood pressure of S-PE patients was decreased significantly after treated with magnesium sulfate combined with compound Danshen injection [24]. In our study, SBP, DBP, D-D, Fib, and 24 h urinary protein in two groups were significantly decreased after treatment. Moreover, PT and APTT were increased in both groups. In patients with S-PE, vasospasm and contraction of liver vessels lead to hypoxic-ischemic injury of liver tissues [25]. The contents of ALT, AST, and other liver function landmark enzymes increased significantly, which can quantitatively reflect the degree of liver function injury and clinical treatment effect. With the increase of urinary protein, the patients' renal function injury was aggravated, followed by glomerular dilatation and renal tubule spasm and gradually decreased renal blood flow and glomerular filtration excess, leading to the increase of BUN and Scr [26]. Our results showed that the levels of ALT, AST, BUN, and Scr in two groups were decreased after treatment, suggesting that the two treatments have the effect of optimizing liver and kidney function. The levels of ALT, AST, BUN, and Scr in the therapy group were lower, indicating that compound Danshen injection-assisted therapy can further improve the liver and kidney functions of patients with S-PE, and its efficacy is more outstanding. Consistent with the previous results [27, 28], our therapeutic effect was better, which was possibly due to the insufficient sample size. In conclusion, compound Danshen injection adjuvant therapy can optimize the clinical manifestations of S-PE and reduce systemic hypercoagulability.

## Figures and Tables

**Table 1:** Comparison of EPCR and TM levels before and after treatment $\left(\bar{x}+s\right)$.

Parameters	Control group		Therapy group	
	Before treatment	After treatment	Before treatment	After treatment
EPCR (*μ*g/L)	217.03 ± 9.41	165.31 ± 13.43	217.96 ± 9.53	142.21 ± 12.41
TM (*μ*g/L)	89.21 ± 8.25	60.97 ± 8.14	89.51 ± 6.67	49.02 ± 8.45

**Table 2:** Comparison of CAT and LPO levels before and after treatment $\left(\bar{x}+s\right)$.

Parameters	Control group		Therapy group	
	Before treatment	After treatment	Before treatment	After treatment
CAT (U/mL)	4.92 ± 0.88	7.34 ± 1.34	4.99 ± 0.94	8.36 ± 1.45
LPO (nmol/L)	14.75 ± 2.31	11.34 ± 2.12	14.88 ± 2.44	9.98 ± 1.98

**Table 3:** Comparison of serum coagulation function indexes before and after treatment $\left(\bar{x}+s\right)$.

Parameters	Control group		Therapy group	
	Before treatment	After treatment	Before treatment	After treatment
Fib (g/L)	3.93 ± 0.42	3.19 ± 0.33	3.96 ± 0.45	2.89 ± 0.29
D-D (mg/L)	2.64 ± 0.33	2.12 ± 0.27	2.63 ± 0.36	1.66 ± 0.24
PT (s)	10.18 ± 1.54	11.91 ± 1.74	10.19 ± 1.57	13.31 ± 1.81
APTT (s)	31.10 ± 3.54	33.02 ± 3.71	31.19 ± 3.47	35.01 ± 3.87

**Table 4:** Comparison of liver and kidney function indexes before and after treatment $\left(\bar{x}+s\right)$.

Parameters	Control group		Therapy group	
	Before treatment	After treatment	Before treatment	After treatment
ALT (U/L)	34.71 ± 3.51	26.61 ± 3.09	34.77 ± 3.68	21.78 ± 2.83
AST (U/L)	45.85 ± 5.62	31.79 ± 4.23	45.73 ± 5.41	25.47 ± 2.98
BUN (mmol/L)	6.20 ± 0.64	4.02 ± 0.47	6.18 ± 0.58	3.30 ± 0.41
Scr (*μ*mol/L)	323.73 ± 43.77	280.74 ± 34.27	321.99 ± 43.59	22.38 ± 31.87

## Data Availability

The data used to support the findings of this study are available on reasonable request from the corresponding author.
